# Case Report: Analysis of colposcopic image features in a case of early-stage small cell neuroendocrine carcinoma of the cervix

**DOI:** 10.3389/fonc.2025.1653318

**Published:** 2026-01-12

**Authors:** Leilei Yuan, Kejie Liu

**Affiliations:** Department of Gynecology, Fuyang People’s Hospital (The Affiliated Fuyang People’s Hospital of Anhui Medical University), Fuyang, Anhui, China

**Keywords:** cervical small cell neuroendocrine carcinoma, cervical cancer, colposcopy images, renal ectopia, early-stage

## Abstract

This study analyzes the colposcopy findings of an early-stage cervical small cell neuroendocrine carcinoma with ectopic kidney in a patient treated at Fuyang Peoples Hospital in October 2023. The colposcopic findings primarily included a well-defined, dense, thickened cervix with atypical and fragile blood vessels, along with irregular stromal infiltration. Pathological examination confirmed cervical small cell neuroendocrine carcinoma. The patient underwent a transabdominal radical hysterectomy with bilateral salpingectomy, pelvic lymph node dissection, and bilateral ovarian repositioning on October 5, 2023. No adjuvant therapy was administered postoperatively, and follow-up over 15 months showed no evidence of disease recurrence or metastasis. The study highlights the critical role of colposcopy in early detection of cervical small cell neuroendocrine carcinoma and its significant impact on improving patient outcomes.

## 1 Introduction

Neuroendocrine neoplasms (NENs) are those that originate from peptide neurons Neuroendocrine cells, tumors with expression of neuroendocrine markers, are rare and can occur in various parts of the body, most commonly in the lungs and the gastric and pancreatic sections, but rarely in the female reproductive system (1) Female reproductive system NEN usually occurs in the cervix and ovaries (2). In the 5th edition of WHOs classification of gynecological tumors in 2020, NEN was divided into neuroendocrine neoplasms (NET) and neuroendocrine carcinomas (NEC), the latter of which were divided into small cell neuroendocrine carcinoma and large cell neuroendocrine carcinoma. NEC was the rarest (Rindi et al., 2018), but the cervix is a common site for NEC in the female reproductive system (3). Cervical small cell neuroendocrine carcinoma accounts for about 0.9%-1.1% of cervical cancer (4), Cervical small cell neuroendocrine carcinoma (SCNEC), a highly aggressive subtype of cervical malignancy, frequently metastasizes through lymphatic channels during early stages and demonstrates poor prognosis with high recurrence rates. For early-stage patients with tumors ≤4 cm, the 5-year survival rate ranges from 30% to 46%, whereas advanced cases exceeding 4cm show a significantly lower 5-year survival rate of 0-15% (5). At present, there are many early SCNEC related reports at home and abroad (6), However, a comprehensive review of domestic and international literature revealed no existing reports on early-stage SCNEC (Small Cell Neuroendocrine Carcinoma of the Cervix) colposcopy imaging features.

## 2 Case report

This case report presents a clinical analysis of an early-stage cervical small cell neuroendocrine carcinoma with renal ectopia. The colposcopy terminology used in this study adheres to the 2011 terminology established by the International Federation for Cervical Pathology and Colposcopy (IFCPC) (7). (including atypical blood vessels, fragile blood vessels, irregular lesion surfaces in suspicious cancer cases, and high-grade lesions characterized by dense thickening, coarse inhomogeneous stromal infiltration, and sharp lesion margins), combined with the red zone (highly vascularized areas) defined in Chinas R-way colposcopy evaluation system. The latter demonstrates near-100% cumulative sensitivity for identifying cervical HSIL + (8). The colposcopy images of the patient were described, and the characteristics of early cervical small cell neuroendocrine carcinoma colposcopy images were observed to identify cervical small cell neuroendocrine carcinoma as early as possible, so as to improve the prognosis and survival rate of patients.

### 2.1 General information

The patient, a 34-year-old female, sought treatment at Fuyang Peoples Hospital Gynecology Department on October 5, 2023 for “vaginal bleeding for over seven months following intercourse.” Her last menstrual period was recorded on September 14, 2023. She developed post-intercourse vaginal bleeding in February 2023 but did not take it seriously. On July 20, 2023, she visited a local clinic due to persistent symptoms. Cervical liquid-based cytology examination revealed atypical squamous epithelial cells with possible high-grade squamous intraepithelial neoplasia (ASC-H), while high-risk human papillomavirus (HPV) type 18 testing was positive. She returned to our department on August 26, 2023.

The patient had no prior medical history, had not undergone regular cervical cancer screening, and was unvaccinated against HPV. She denied having hypertension, diabetes, or other conditions, and maintained no smoking or alcohol consumption. She became a mother at age 20 with two pregnancies and two deliveries, and her father had died of lung cancer. Gynecological examination findings: Vulva: Postpartum; Vagina: Unobstructed with smooth mucosa; Cervix: Mild erosive changes, no significant space-occupying lesions, soft texture; Uterus: Anteverted, usually enlarged, mobile, non-tender; Adnexa: No palpable abnormalities in bilateral adnexal regions; Bimanual examination: No detectable thickening beside the uterus, no blood-stained glove.

### 2.2 Check up

**Figure 1:**
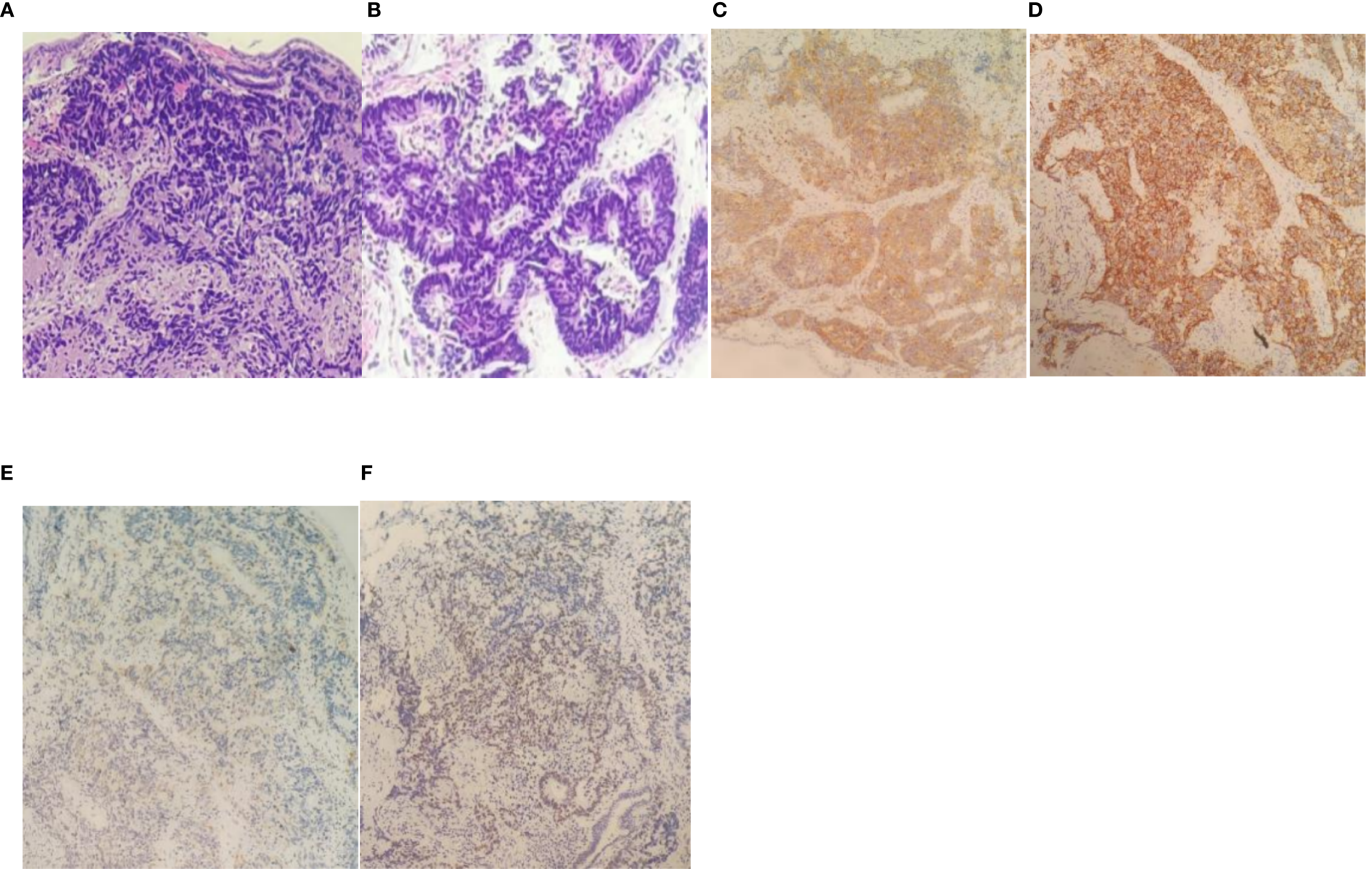
Pathological examination. **(A)** HE staining (×10) shows patchy infiltration of small cell neuroendocrine tumor cells beneath the cervical epithelium, accompanied by patchy necrosis. **(B)** HE staining (×40) reveals indistinct cell borders, scant cytoplasm, irregularly shaped nuclei with angular projections, fine and diffuse chromatin appearing dust-like, indistinct nucleoli, and numerous mitotic figures under high magnification. **(C)** Immunohistochemistry shows Syn-positive expression (EnVision method ×200). **(D)** Immunohistochemistry demonstrates CD56-positive expression (EnVision method ×200). **(E)** Immunohistochemistry reveals CgA-positive expression (EnVision method ×200). **(F)** Immunohistochemistry shows TTF-1 positive expression (EnVision method ×200).

**Figure 2:**
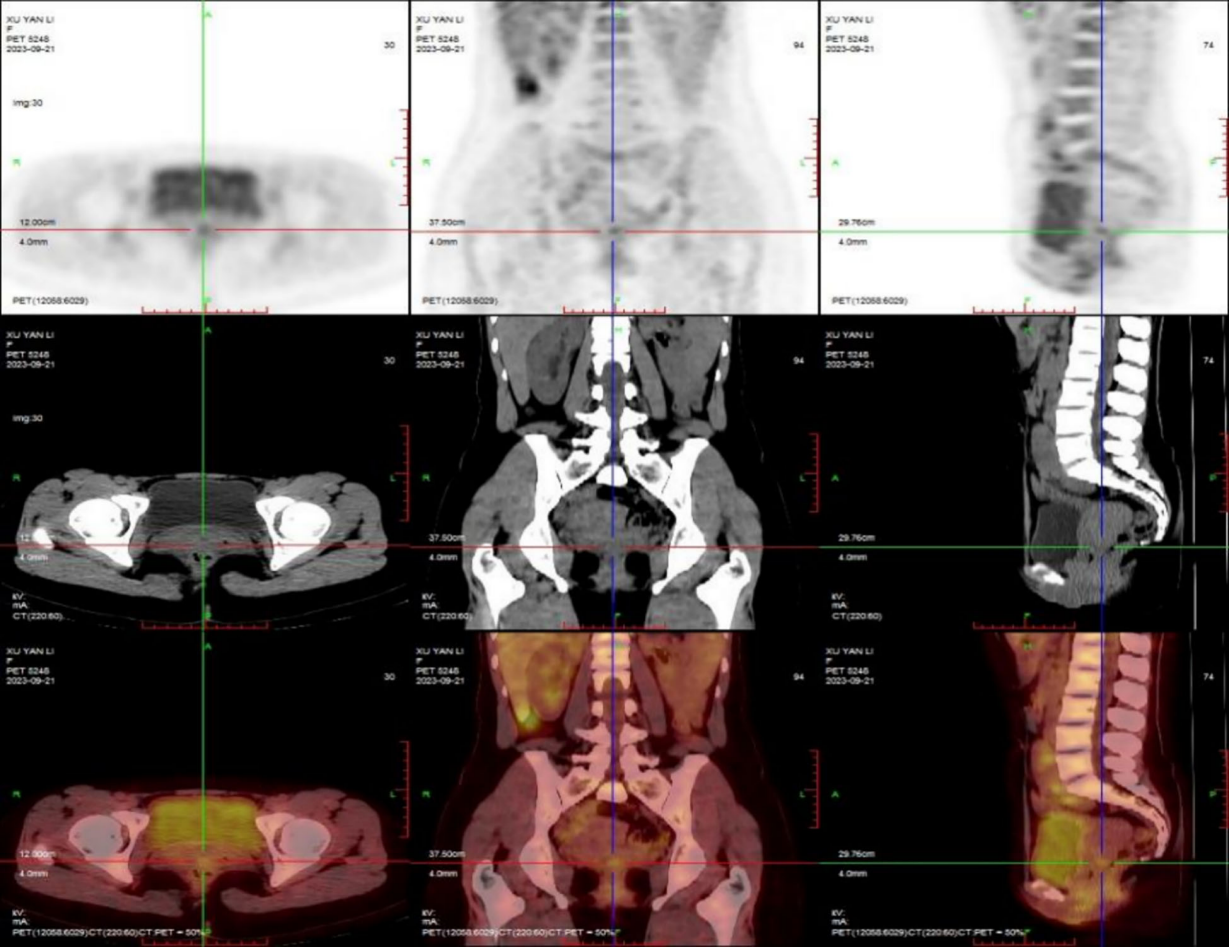
PET-CT imaging shows slightly thickened soft tissue in the cervix and increased FDG metabolism. The “cross” intersection in the figure is the lesion site.

**Figure 3:**
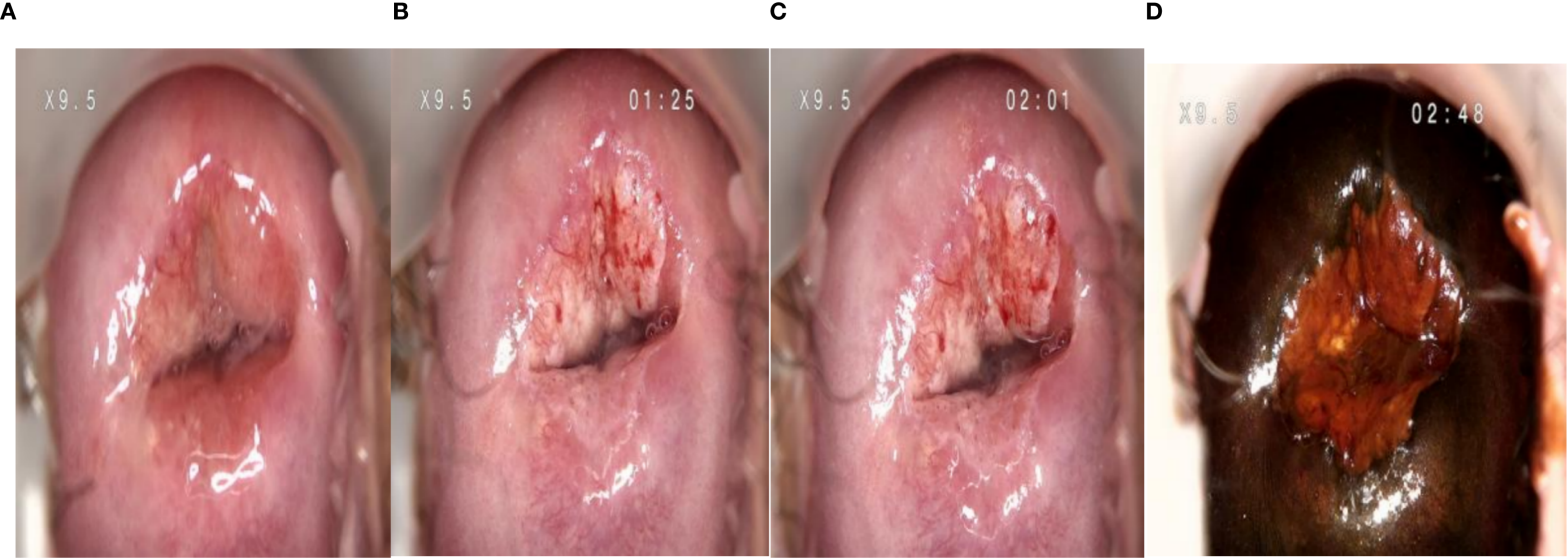
Colposcopy images. **(A)** After gently wiping the cervical surface secretions with saline-soaked cotton balls, images were captured (Figure **A** shows an irregular surface at the 12 oclock position of the anterior lip with grayish-yellow coloration and abundant blood supply). **(B)** After 50 seconds of 5% acetic acid treatment, images were taken at 1 minute and 25 seconds post-treatment. **(C)** Similarly, images were captured at 1 minute and 25 seconds after 50 seconds of 5% acetic acid application (Figures **B**, **C** demonstrate persistent whitening of the anterior lip with coarse inhomogeneous striae and atypical fragile vessels following continuous 5% acetic acid exposure). **(D)** Images were obtained after applying Rous iodine solution to the cervix (Figure **D** shows non-staining of the anterior lip with a mustard-yellow appearance).

**Figure 4:**
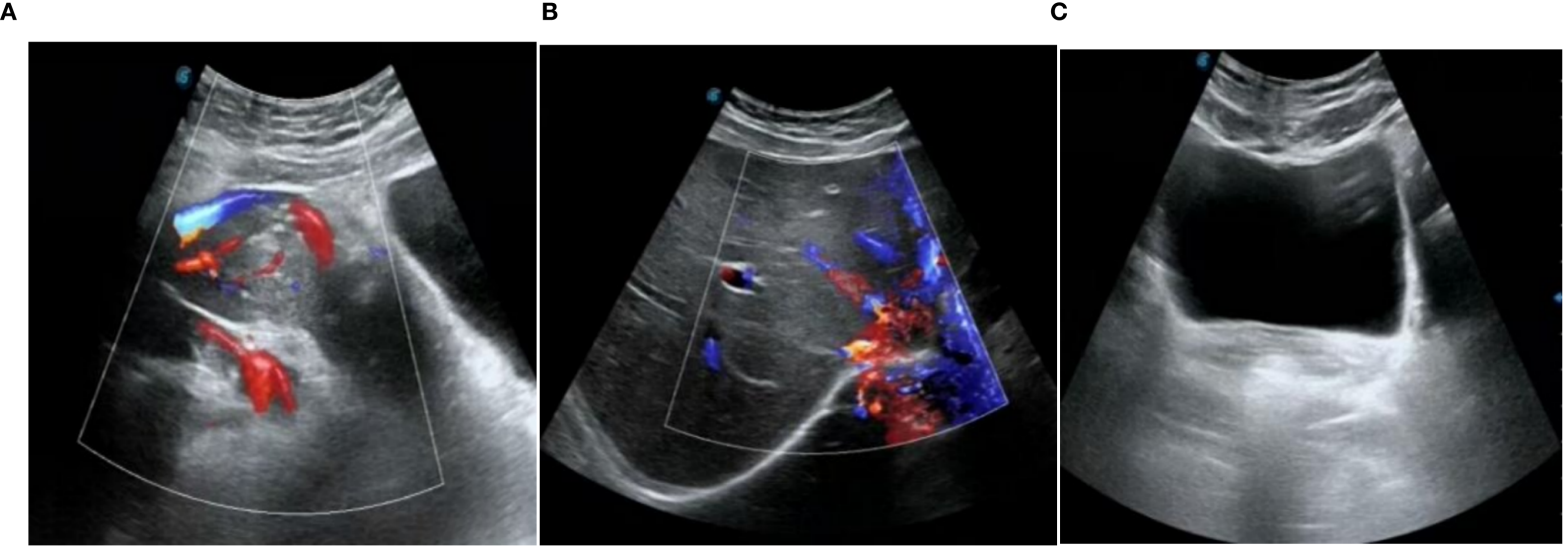
Pelvic color ultrasound and liver, gallbladder, pancreas, spleen and kidney color ultrasound at 15 months after surgery. **(A)** pelvic ectopic kidney image **(B)** liver image **(C)** vaginal stump image.

Vaginal speculum examination findings on August 26, 2023: Under 5% acetic acid application, well-demarcated dense white lesions with thickened margins were observed in the first and fourth cervical quadrants. Coarse irregularities and small necrotic foci were noted at the 12 oclock position of the cervix, accompanied by atypical and fragile blood vessels. The colposcopy diagnosis suggested suspected cervical invasive carcinoma (see **Figures 1**–**4**).

Cervical biopsy specimens from points 1, 11, and 12 were pathologically diagnosed with high-grade neuroendocrine carcinoma (predominantly small cell neuroendocrine carcinoma components, with focal presence of small cell neuroendocrine carcinoma components). Immunohistochemical results: Tumor cells showed CKpan (+), Vim (-), ER (-), PR (-), CEA (+), Syn (+), CgA (focal +), CD56 (+), SMARCA4 (moderate +), P16 (diffuse +), P53 (-), CK7 (-), CK20 (-), TTF-1 (moderate +), and Ki-67 (+ with hotspots covering 90% of the area).

On September 21, 2023, the PET-CT imaging at our hospital revealed: 1. Slight thickening of soft tissue in the cervix with slightly elevated FDG metabolism (see **Figure 2**). Combined with the patients history of cervical malignancy, no abnormal metastatic signs of FDG metabolism elevation were observed in the remaining trunk and brain regions; 2. Bilateral thyroid with heterogeneous density and slightly elevated FDG metabolism, suggesting benign lesions; 3. Left kidney ectopia and minor uterine fluid accumulation.

### 2.3 Diagnose

Diagnosis: 1. Early cervical small cell neuroendocrine carcinoma 2. Ectopic kidney.

### 2.4 Treatment and follow-up

On October 9, 2023, the patient underwent a radical hysterectomy with bilateral salpingectomy, pelvic lymphadenectomy, and bilateral ovarian repositioning. The intraoperative findings revealed a significantly enlarged uterus, with no abnormalities observed in the bilateral adnexal structures. The liver, spleen, gastrointestinal tract, diaphragm, intestines, omentum, and peritoneal surface showed no abnormalities. An ectopic left kidney was identified anterior to the sacrum, while paraspinal lymph nodes remained normal. Following cervical cancer hysterectomy classified by the Q-M staging system, the C1-type resection was performed. The ovaries were elevated and fixed to the ipsilateral posterior abdominal wall, positioned 5cm above the pelvis. Postoperative pathology confirmed: 1. “Cervical” malignant tumor: Histological and immunohistochemical analysis indicated endocrine cell carcinoma of high-grade endophytic infiltration. The tumor infiltrated approximately 0.5cm in depth and 0.9cm in width, with vascular invasion (+) but no confirmed nerve invasion. No cancerous tissue was found in the cervical canal or endometrium, vaginal fornices (frontal and posterior), bilateral fallopian tubes, or uterine parametrium, though the vaginal wall margin was negative. Bilateral fallopian tube mesosalpinx cysts were present. 2. “Right pelvic lymph nodes” (negative, 0/8).3. “Left pelvic lymph nodes” (negative, 0/11). Based on microscopic examination of lesion size, postoperative diagnosis included cervical small cell neuroendocrine carcinoma IA2 stage and ectopic kidney. Postoperative radiotherapy and chemotherapy were recommended. During radiation oncology consultation, clinicians considered the left renal ectopia anterior to the sacrum and proposed pelvic external beam radiation combined with intravaginal brachytherapy, which would significantly impact the leftRegarding renal function, adjuvant chemotherapy was recommended, but the patient declined. Follow-up examinations in June 2024 showed negative results for TCT and HPV, with chest CT scans, squamous epithelial carcinoma antigen (SECA) tests, pelvic ultrasound, and liver, gallbladder, pancreas, spleen, and kidney ultrasounds all demonstrating no abnormalities. Subsequent January 2025 follow-up tests for SECA, pelvic ultrasound, and liver/gallbladder/pancreas/spleen/renal ultrasound also revealed no abnormalities. After 15 months of postoperative follow-up, no recurrence or metastasis of the disease has been detected through relevant examinations (see **Figure 4**). The patients medical history and follow-up process are detailed in **Table 1**.

**Table 1:** Patient diagnosis, treatment and follow-up.

Time	Symptoms and diagnosis and treatment
February 2023	Vaginal bleeding after intercourse
20 July 2023	TCT: ASC-H, HPV18 positive
26 August 2023	Vaginal examination and cervical biopsy
21 September 2023	PET-CT check up
5 October 2023	be in hospital
9 October 2023	The procedure included transabdominal wide hysterectomy, bilateral salpingectomy, pelvic lymph node dissection, and bilateral ovarian transposition
November 15, 2023	Radiotherapy is not recommended in the radiotherapy department because of ectopic kidney (left kidney located in the pelvis)
June 2024	No recurrence or metastasis was found during the first follow-up
January 2025	In the second follow-up, no recurrence or metastasis was found

## 3 Discussion

### 3.1 Etiology and pathogenesis of cervical small cell neuroendocrine carcinoma

Small cell neuroendocrine tumors can occur in any female reproductive organ, most commonly in the cervix, but are rare and have a low incidence among cervical malignancies (1) Persistent infection with high-risk HPV types is the primary cause of cervical cancer and its precancerous lesions. The development of cervical SCNEC (cervical squamous cell neoplasia) is also closely associated with high-risk HPV infections. In nearly all cases of high-grade cervical neuroendocrine carcinoma, high-risk HPV strains are detected, with types 16 and 18 being the most common. Notably, type 18 is more prevalent than type 16 (9, 10). This demonstrates that early HPV vaccination can effectively prevent disease development. The patient in this report was co-infected with HPV18, which exhibits distinct pathogenic mechanisms compared to other cervical malignancies. Cervical squamous cell carcinoma and adenocarcinoma result from high-risk HPV types acting on basal reserve cells of the cervical epithelium, altering their genetic structure and ultimately leading to cancerous transformation. Molecular biological studies on cervical neuroendocrine carcinoma have revealed that (11, 12) Cervical SCNEC has a high frequency of 3-chromosome short arm heterozygous deletion, which is similar to the pathogenesis of small cell lung cancer. The common gene mutation sites are PIK3, KRAS, and TP53. However, we still need more research data to further explore the pathogenesis of cervical small cell neuroendocrine carcinoma.

### 3.2 Pathological diagnosis of cervical small cell neuroendocrine carcinoma

The diagnosis of cervical SCNEC primarily relies on pathological morphology and immunohistochemical detection methods. Morphologically, it resembles pulmonary neuroendocrine carcinoma. The neuroendocrine tumor markers for cervical SCNEC include chromaffin globulin (CgA), CD56, neuron-specific enolase (NSE), synaptin (Syn), and thyroid transcription factor-1 (TTF-1). Among these, CD56 is the most sensitive neuroendocrine marker, while CgA serves as the most specific indicator (13, 14). The immunohistochemical results of this study showed Syn (+), CgA (focal +), CD56 (+), and TTF-1 (few +), which were consistent with the above studies and consistent with the diagnosis of cervical small cell neuroendocrine carcinoma.

### 3.3 Treatment, prognosis and follow-up of cervical small cell neuroendocrine carcinoma

According to the 2025 U.S. National Comprehensive Cancer Network Guidelines for Cervical Cancer (15), The small cell NECC tumor was confined to stage IA1-IB2 cervical cancer. The treatment protocol included radical hysterectomy with pelvic lymph node dissection± para-aortic lymph node sampling, followed by postoperative chemotherapy or concurrent chemoradiotherapy. The patient received preoperative diagnosis, as the small cervical lesion was difficult to visualize with naked eye and PET-CT scans showed no metastasis. Given the patients desire to preserve fertility, diagnostic cervical conization was omitted in favor of immediate radical surgery. Currently, there is no standardized chemotherapy regimen for cervical SCNEC. Due to the high similarity between cervical SCNEC and small cell lung cancer (SCLC) in terms of biological behavior and gene-protein expression profiles, treatment plans reference SCLC protocols, typically using cisplatin (carboplatin as an alternative if cisplatin is contraindicated) combined with etoposide (EP) (16, 17). Domestic scholars have shown that chemotherapy can improve the prognosis of cervical SCNEC patients, while radiotherapy does not significantly improve the prognosis of early-stage patients (18). In addition, the patients left kidney was ectopic to the pelvis and happened to be located in the pelvic external radiation target area. Radiotherapy would lead to vascular changes in the left kidney, thus affecting renal function (19). And studies have found that surgery and chemotherapy can improve the prognosis of cervical small cell neuroendocrine carcinoma patients (20), Therefore, adjuvant chemotherapy is recommended after surgery, but the patient refused to receive adjuvant chemotherapy for personal reasons. Some studies have also found that high risk factors for recurrence and metastasis of cervical SCNEC include lymphatic vascular infiltration (LVSI) positivity and small cell component proportion (21). This study demonstrates that cervical small cell neuroendocrine carcinoma (SCNEC) exhibits lymphatic and vascular invasion at a mere 5mm depth of tumor infiltration, highlighting the diseases high aggressiveness. Notably, Zhu Yuan et al. established that FIGO staging (2018 edition) serves as a key prognostic factor for SCNEC patients, with earlier-stage cases showing better clinical outcomes (17). Early detection and timely treatment are crucial to improve patient prognosis and prolong survival. Currently, there is no specific follow-up protocol for cervical small cell carcinoma. The content and intervals of follow-up still adhere to the guidelines outlined in the 2025 NCCN Clinical Practice Guidelines for Cervical Cancer (15). No recurrence or metastasis has been observed during the 15-month follow-up period. This outcome may be attributed to two factors: cervical SCNEC lesions being small and microscopically detectable, allowing for early surgical intervention without chemotherapy, which demonstrates comparable efficacy to postoperative chemotherapy. Furthermore, the procedure was performed by surgeons with extensive experience, and strict tumor-free concepts during surgery reduced recurrence risks. While the relatively short follow-up duration also contributed to this result, studies indicate that patients with Stage I SCNEC receiving treatment achieved a median progression-free survival (PFS) of 15.4 months (22).

### 3.4 Endoscopic image characteristics of cervical small cell neuroendocrine carcinoma

A comprehensive review of domestic and international literature reveals no existing reports on colposcopy imaging manifestations of early-stage cervical small cell neuroendocrine carcinoma (SCNEC). While grossly visible lesions at stage IB and above can be readily detected through routine gynecological examinations, IA-stage SCNEC requires colposcopy for microscopic identification. As established in oncology, tumor development originates from genetic alterations in cellular structures that disrupt normal regulatory mechanisms, leading to abnormal proliferation. During rapid tumor growth, characteristic features include abundant neovascularization with enhanced blood supply, along with increased vascular fragility that predisposes to hemorrhage and necrosis. These pathological processes similarly characterize cervical malignancies. Therefore, the R-way colposcopy diagnostic system is specifically designed to address these pathophysiological characteristics (23). The areas with abundant blood supply observed under colposcopy include columnar epithelium, immature metaplastic regions, and abnormal tissue areas. The red zone (R zone) of this system represents the most prominent red area outside the columnar epithelial region. Cervical columnar epithelium is a single-layered structure with abundant subepithelial vessels. However, when treated with 5% acetic acid, the columnar epithelium exhibits characteristic features under colposcopy, appearing as “villous, “ red granular, or grape-like formations (23). In this way, the location of the red zone can be clearly defined. Abnormal cervical epithelial cells are monoclonal hyperplasia, with increased nuclear surface area and increased nucleus-to-sphere ratio. After 5% acetic acid action, the nucleoprotein coagulates and obstructs light penetration, thus appearing as a dense thick white color with sharp boundary in the red zone (23). The characteristics of small cell neuroendocrine tumor cells are a large number of small cell aggregation, large nucleus, deep staining, little cytoplasm, increased nuclear ratio, not obvious nucleolus, accompanied by necrosis (24), The acetate-induced dense white lesion indicates tumor growth requiring neovascularization for nourishment. This results in red vascular areas under colposcopy, characterized by fragile blood vessels lacking smooth muscle that bleed easily. The disordered arrangement of these vessels appears as irregularly enlarged mosaic patterns, accompanied by atypical blood vessels and hemorrhages – a hallmark manifestation of severe pathological changes (25, 26). The saline-vaginal speculum images in this study revealed well-vascularized areas on both anterior and posterior cervical lips. The red zone was located on the anterior lip, where at 12 oclock on the green light image, atypical blood vessels were observed around the cervical concave area. After 50 seconds of application of 5% acetic acid, no new SCJ was visible on the anterior lip, while new SCJ appeared on the posterior lip. Persistent thick whiteish discoloration with coarse irregular patterns was noted in the red zones of the first and fourth quadrants of the anterior lip, along with fragile blood vessels and atypical vascular patterns at the 12 oclock position. Therefore, the lesion in this report primarily involved the anterior cervical lip, with more severe involvement occurring at the 12 oclock cervical region. The reaction between Rous iodine solution and glycogen within cervical epithelial cells … (23) The lesion appears brownish-black, with mature squamous epithelial cells rich in glycogen that shows no coloration after iodine staining. In contrast, cervical intraepithelial neoplasia (CIN), invasive carcinoma, or immature metaplasia contain little to no glycogen and remain non-staining. The thick white area on the anterior lip of the cervix in this case demonstrates “mustard-yellow” staining without iodine reaction. The red zone exhibits thick white discoloration, coarse irregular mosaic patterns, atypical blood vessels, and fragile capillaries, suggesting endoscopic suspicion for invasive carcinoma. While the patients endoscopic findings resemble high-grade cervical intraepithelial neoplasia (e.g., thick white discoloration, sharp margins, and irregular mosaic patterns) – which could be mistaken for early-stage invasive carcinoma with concurrent high-grade lesions – histopathological examination ruled out high-grade squamous intraepithelial neoplasia. Notably, a regular-angled depression with grayish-yellow surface is observed at the 12 oclock position, differing from tumor necrotic ulcers characterized by irregular margins, uneven surfaces, and deep myofascial invasion. Does this imaging feature represent an early hallmark of SCNEC? Further data support is required.

The early detection of cervical small cell neuroendocrine carcinoma (SCNEC) reported in this study is attributed to three key factors: 1. Cytopathologists identify highly abnormal cells, providing critical evidence for subsequent diagnosis and treatment; 2. Specialist colposcopy physicians can precisely locate the most severe cervical lesions and perform biopsy procedures; 3. Pathologists possess specialized expertise and extensive experience in diagnosing SCNEC, enabling accurate disease identification.

### 3.5 Shortcomings

A literature review of cervical small cell neuroendocrine carcinoma (SCNEC) both domestically and internationally revealed no existing reports on its colposcopy imaging characteristics. Given the low repeatability of colposcopy examinations and the distinct colposcopic features observed in different SCNEC subtypes (endogenous, exophytic, ulcerative, etc.), we need to collect more data for further research and summarization to enable early detection of precancerous lesions and improve patient prognosis. Additionally, the patient was incidentally diagnosed with isolated renal ectopia. Current literature shows no documented cases of SCNEC coexisting with renal ectopia. Is there a potential correlation between this condition and SCNEC? Or could there be associated genetic mutations? Of course, these are just hypotheses that require scientific evidence to substantiate.

## Data Availability

The original contributions presented in the study are included in the article/supplementary material. Further inquiries can be directed to the corresponding author.
